# Prevalence of low back pain, seeking medical care, and lost time due to low back pain among manual material handling workers in the United States

**DOI:** 10.1186/s12891-019-2594-0

**Published:** 2019-05-22

**Authors:** Sue A. Ferguson, Andrew Merryweather, Matthew S. Thiese, Kurt T. Hegmann, Ming-Lun Lu, Jay M. Kapellusch, William S. Marras

**Affiliations:** 1 Spine Research Institute, The Ohio State University, 210 Baker Systems, 1971 Neil Avenue, Columbus, OH 43210 USA; 2 Rocky Mountain Center for Occupational & Environmental Health, Department of Family and Preventive Medicine, University of Utah, 391 Chipeta Way, Suite C, Salt Lake City, UT 84108 USA; 3 National Institute for Occupational Safety and Health, Taft Laboratories, 1090 Tusculum Ave. MS C-24, Cincinnati, OH 45226 USA; 4 Occupational Science & Technology, University of Wisconsin-Milwaukee, P.O. Box 413, Milwaukee, WI 53201 USA

**Keywords:** Low back pain, Prevalence, Seeking medical care, Lost time

## Abstract

**Background:**

Low back pain (LBP) is a common and costly problem throughout the United States. To achieve a greater understanding of the occupational risk factors, the National Institute for Occupational Safety and Health (NIOSH) funded a low back health effects consortium, which performed several surveillance studies throughout the United States. This study combines data from the consortium research groups resulting in a data set with nearly 2000 workers in various regions of the country. The purpose of this paper is to examine prevalence and personal risk factors of low back health effects among these workers.

**Methods:**

There were three common questions regarding history of low back health effects in the past 12 months including 1) have you had LBP lasting 7 days, 2) have you sought medical care for LBP, and 3) have you taken time off work due to LBP. The questionnaire included demographic questions. There were five data collections institutions or sites including NIOSH, Ohio State University, University of Wisconsin-Milwaukee, Texas A&M University, and University of Utah.

**Results:**

The 12-month period prevalence of low back pain lasting 7 days, seeking medical care, and lost time due to LBP were 25, 14 and 10%, respectively. There were no statistically significant differences in gender, age or weight between cases and non-cases for any prevalence measure. The height of workers was significantly greater in the cases compared to non-cases for all three prevalence definitions. There were significant differences among the sites on the prevalence of seeking medical care for LBP and lost time due to LBP. The Ohio State University had significantly higher prevalence rates for seeking medical care and lost time due to LBP than University of Wisconsin, University of Utah, or Texas A&M University.

**Conclusion:**

LBP, the least severe low back health effect studied, had the highest prevalence (25%) and lost time due to LBP, the most severe low back health effect studied, had the lowest prevalence (10%) among nearly 2000 US manual material handling workers. There was a significant site or regional influence in prevalence rates for seeking medical care and lost time due to LBP.

## Background

Low back pain is a leading cause of disability worldwide [1]. There are several surveillance measures that have been used in the literature to investigate prevalence rates and risk factors for low back health effects including presence of LBP of any severity, LBP that resulted in the seeking of medical care, and LBP resulting in lost time from work. Reported LBP prevalence rates range from 4% [2] to 69% [3, 4] and vary depending on the length of time evaluated (e.g., lifetime, 1-month and point prevalence [5] as well as pain intensity [6]). LBP resulting in seeking medical care has reported prevalence rates ranging from 4.5% [7] to 32% [8] and has been shown to be influenced by the length of time of symptoms [9], gender [10] and race/ethnicity [10]. Taking time off from work, or lost time, is the least commonly used surveillance measure for low back health effects in the literature [11] and has prevalence rates between 4.6% [12] to 18% [13, 14]. These surveillance measures for low back health may represent a series of cascading events that start with mild LBP, which perhaps leads to an individual seeking medical care for LBP, possibly progressing to taking time off work for LBP that may recur any number of times and culminate with disabling LBP [15]. It is theorized that evaluating these three surveillance measures in a large population may provide further insight into the progression and potential prevention of LBP leading to disability.

A few studies have examined prevalence rates among workers using surveillance measures of LBP, seeking medical care for LBP and lost work time due to LBP. Ozgular et al. [6] examined prevalence of LBP in 725 active workers (office, hospital, warehouse, and airport registration of luggage) using LBP lasting at least 1 day, LBP with health care professional visit(s), and LBP with the taking of sick leave. The results showed a 1 day prevalence of 43%, seeking medical care prevalence rate of 22%, and LBP with sick leave of 9% [6]. Merlino et al. [16] examined musculoskeletal disorders among 996 apprentice construction workers and found that in the past 12 months the prevalence for LBP symptoms was 54%, seeking medical care prevalence was 17%, and missed work prevalence was 7%. Feng et al. [17] evaluated a group of 244 Taiwanese nurses and nursing staff and reported 66% prevalence of LBP lasting at least 1 day, 38% seeking medical care for LBP, and 11% with sick leave for LBP in the past 12 months. Abolfotouh et al. [13] examined several low back prevalence measures among a population of 254 nurses in Doha, Qatar. The 12 month period prevalence for LBP lasting at least 1 day was 54%, prevalence for seeking treatment due to LBP was 34%, and sick leave or lost time for LBP prevalence was 18% [13].

Thus, there is a wide range of prevalence rates for low back health effects. These prevalence rates may be influenced by the length of time as well as the severity of low back health effect definition investigated, i.e. LBP, seeking medical care for LBP or lost work time for LBP. There is a lack of studies examining the prevalence of low back health effects using multiple definitions in a large occupational population in the United States. The objective of this study was two-fold 1) to combine data from several studies and examine the differences in prevalence of LBP, seeking medical care for LBP, and lost time due to LBP among a large population of US manual material handling workers and 2) to examine differences among personal risk factors for each prevalence measure.

## Methods/design

This was a cross-sectional study design that combined data from several epidemiological studies that examined work related low back health. It is hypothesized that there will be differences in the prevalence rates among the three definitions of LBP furthermore this will result in differences among personal risk factors for the prevalence measures. The LBP research consortium included 5 members: 1) National institute of Occupational Safety and Health (NIOSH) 2) Ohio State University (OSU), 3) University of Wisconsin-Milwaukee (UWM), 4) Texas A&M University (TAMU), and 5) University of Utah (UU). The individual consortium study findings have been previously published [5, 18–20]. All studies were approved by the respective institutional review boards (IRBs). The University of Wisconsin IRB had oversite for the merging of anonymized datasets.

Convenience samples of workers were enrolled from six US states: Illinois, Michigan, Ohio, Texas, Utah and Wisconsin. The NIOSH data was collected at a large dryer manufacturing facility in the state of Ohio. The OSU team collected data in 20 distribution centers in Ohio and 1 office furniture distribution center in Michigan. The 20 distribution centers were in one of four categories including automotive parts, grocery, apparel and general merchandise. The UU data was collected in the state of Utah in a variety of manufacturing facilities including medical products, apparel, cabinetry, salt and auto parts manufacturers as well as an alcohol distribution center and printing operations. The TAMU group collected data primarily at meat packing and grocery warehouse facilities and one upholstery facility in the state of Texas. The UWM data was collected in 15 facilities in the state of Wisconsin and one facility in the state of Illinois, and consisted of manufacturing and distribution facilities including automotive, apparel, window and door, food and beverage, household items, pharmaceutical and general merchandise. At all sites the inclusion criterion was the manual material handling job at the facility that the workers performed. Workers on a job of interest were recruited by a research team member. Researchers encouraged all workers on the job of interest to participate regardless of health issues. The exclusion criterion was working at the facility on a job without manual material handling tasks of interest. All workers signed a human subject’s consent form prior to participating in the study. All data was collected at the respective work facilities resulting in a variety of occupational study settings. Participating employees received their regular wages during the study. All workers participating in the study received questionnaires that included demographic information (e.g., age, gender, race/ethnicity, height, weight) and cigarette usage. The questionnaires also included questions about low back health effects due to participants’ jobs. Table 1 lists the specific questions regarding low back health effects.

**Table 1:** Research questions utilized to measure low back health effects

Questions for low back health effect.	
1. In the past 12 months, have you had back pain every day for a week (7 days) or more?	
2. In the past 12 months, how many times have you seen a doctor, nurse, physical therapist or chiropractor or other health care provider for your back symptoms?	
3. How many days have you missed work in the past 12 months because of back symptoms?	

The merged database had three categories of variables including 1) health effects and personal risk factors, 2) psychosocial exposure and 3) physical exposure measures. This analysis was only on prevalence of low back health effects and personal risk factors. Only subjects with complete baseline data for health effects and personal factors were eligible for prevalence analysis. Data analyses were initiated for the seeking of medical care question by dichotomization into either seeking medical care or not seeking medical care. Similarly, the missed work question was dichotomized into either no missed workdays or missed workdays. Statistical analyses were performed using SAS version 9.2. Descriptive statistics were calculated for the number of cases for each low back health effect definition and demographic information. To test for differences in the prevalence rates among the three outcome measures McNemar’s Tests were performed. For continuous measures of age, weight, and height, T-tests were run for each case definition. To examine risk factor differences among the three prevalence measures general linear models (proc GLM) were utilized. Test statements were employed to evaluate differences due to consortium study site, gender, race/ethnicity, and site by race/ethnicity interaction. Post hoc Ryan-Einot-Gabriel-Welsch REGWQ multiple range tests were used to determine significant differences among the sites and race/ethnicity.

## Results

The combined total sample from all the consortium sites was 1976 workers and, of those, 1929 completed the minimum baseline data requirements for inclusion in the prevalence analysis. Table 2 lists total population, cases, and prevalence for the three measures of low back health effects. Prevalence rates for LBP lasting at least 1 week, seeking medical care, and lost time due to LBP were 25, 14, and 10%, respectively. McNemar’s test statistics for LBP lasting 1 week vs. seeking medical care was 211.5 and *p*-value < 0.0001. Prevalence of low back pain lasting at least 1 week vs. lost time due to LBP was a McNemar’s test statistic of 242.5 and p-value < 0.001. Finally the McNemar’s test statistic for seeking medical care vs. lost time due to LBP was 31.1 and *p*-value < 0.0001. Thus, the null hypothesis that the distributions are the same was rejected and therefore, each prevalence definition resulted in a significantly different prevalence rate for the same 1 year period.

**Table 2:** Cases and prevalence as a function of low back health effect definition

	Low Back Health Effect Definition		
	Low Back Pain lasting at least one week	Seeking Medical Care for LBP	Lost Work Time for LBP
Cases	483	272	192
Population of Workers	1929	1927	1929
Prevalence	25%	14%	10%

Seventy-five percent of the participants were male. The average age, weight, and height of the study population are listed in Table 3. The race/ethnicity of the 1929 workers was 1253 (65%) white, 275 (14%) Hispanic/Latino, 269 (14%) black/African American, 52 (3%) Asian, 47 (2%) Native Hawaiian or Pacific Islanders, and 3 (< 1%) Native American or Native Alaskan and 30 (2%) declined to respond or were other. Nearly half of the 1929 (*n* = 956, 49%) of the workers had never smoked.

**Table 3:** T-test results for continuous demographic measures means (standard deviation) between cases and non-cases by low back health effect definition

	Age (years)	Weight (kg)	Height (cm)
Total Sample	36.2 (11.6)	84.8 (20.9)	173.2 (10.2)
Low Back Pain ≥7 days			
Cases	36.6 (11.7)	85.2 (19.5)	174.5 (9.6)
Non Cases	36.1 (11.5)	84.6 (21.4)	172.7 (10.4)
*p*-values	0.4031	0.4840	0.0004*
Seeking Medical Care for LBP			
Cases	35.8 (10.9)	85.6 (19.0)	175.5 (9.4)
Non Cases	36.3 (11.6)	84.6 (21.2)	172.7 (10.4)
*p*-values	0.5586	0.4715	0.0001*
Lost Work Time for LBP			
Cases	35.0 (10.9)	85.3 (19.0)	175.0 (9.1)
Non Cases	36.4 (11.6)	84.7 (21.1)	173.0 (10.4)
*p*-values	0.1191	0.7409	0.0039*

Note: * indicates statistical significance

Table 3 lists the means and standard deviations for age, weight, and height for cases and non-cases for each definition of low back health effect and indicators for significant differences. There was no difference in age or weight between cases and non-cases. Worker stature was significantly different between the cases and the non-cases for all three prevalence measures. Workers with low back health effects were approximately 2.0 cm taller than the workers without.

**Fig. 1:**
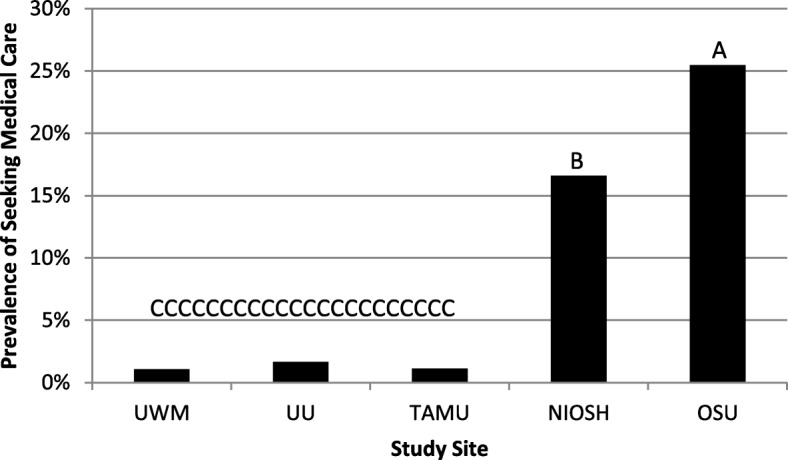
Prevalence of seeking medical care for LBP in the past 12 months as a function of the study site. The bars with different letters above them are statistically significantly difference from one another. Note: UWM = University of Wisconsin, UU = University of Utah, TAMU- Texas A&M University, NIOSH = National Institute of Occupational Safety and Health, OSU = Ohio State University

The general linear model test statement results indicated no significant differences for gender or race for any of the low back health effect prevalence measures. The test statement evaluating differences among the sites varied as a function of the definition of low back health effect. The prevalence measure of LBP lasting at least 1 week showed no statistically significant differences among the sites (data not shown). Conversely, the seeking medical care for LBP prevalence measure showed significant differences among the sites (*p* < 0.001). Figure 1 illustrates the 12-month period prevalence among the different sites. The different letters above the bars in the chart indicate statistically significant differences among the sites from the post hoc REGWQ test results. The OSU site had a significantly greater 12-month period prevalence rate at slightly over 25% than all other sites, as indicated in Fig. 1 by the letter A above the bar. NIOSH had a statistically significant lower 12-month period prevalence of seeking medical care compared to OSU (indicated by the letter B in Fig. 1 above the bar) and a significantly greater 12-month period prevalence in LBP with seeking medical care compared to UWM, UU, and TAMU. There was no difference in seeking medical care 12-month period prevalence among UWM, UU, and TAMU, as indicated in Fig. 1 by the letter C above the bars for the three sites.

**Fig. 2:**
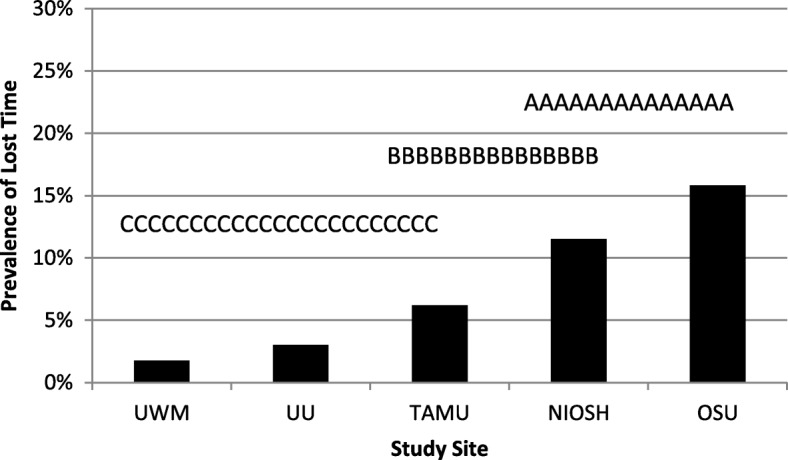
Prevalence of lost time for low back pain in the past 12 months as a function of the study site. The bars with different letters above them are statistically significantly difference from one another. Note: UWM = University of Wisconsin, UU = University of Utah, TAMU- Texas A&M University, NIOSH = National Institute of Occupational Safety and Health, OSU = Ohio State University

The general linear model also showed a statistically significant difference among the sites for lost time due to LBP (*p* < 0.05). Figure 2 illustrates the differences in lost time due to LBP prevalence among the sites. The letters above the bars indicate statistical differences from the post hoc REGWQ test results. The OSU data had the highest 12-month period prevalence at approximately 16% for lost time due to LBP. There was no significant difference between OSU and NIOSH at 12% in lost time as indicated with the letter A above both bars, but OSU had significantly higher lost time 12-month period prevalence than the other three sites. Figure 2 illustrates with the letter B above both bars that there was no significant difference between NIOSH and TAMU at 6% lost time prevalence. Finally, there was no significant difference in lost time prevalence among UWM, UU, and TAMU, as shown in Fig. 2 with the letter C above all three sites.

## Discussion

The principal finding of this research was that prevalence rates of low back health effects in the past 12 month period varied dramatically as a function of the three definitions in a large population of US workers. The prevalence rates for LBP lasting at least 1 week, seeking medical care, and lost time due to LBP were 25, 14 and 10%, respectively. McNemar’s test showed that each of the three prevalence rates were statistically significantly different from one another. Worker stature was the only personal risk factor that was significantly different between cases and non-cases among the three prevalence measures, and that average difference was small (i.e., cases approximately 2 cm taller than non-cases).

One of the strengths of the current study was the nearly 2000 workers that participated from six US states. This is the largest study of its kind that evaluated multiple definitions of low back health effects. Examining the prevalence rates with respect to the theory of cascading events [15], LBP the earliest surveillance measure studied had the highest prevalence, progressing next to medical visits there was a decrease in prevalence and finally to lost time due to LBP which had the lowest prevalence and presumed to be the most advanced or severe low back health effect in the progression. Thus, as surveillance measures for low back health effects progress in severity the prevalence rates decrease. Examining risk factors for each of these surveillance measures may provide insights into the prevention of chronic disabling LBP.

The pattern of period prevalence rates in the current study may be compared to other studies that examined all three prevalence measures. The current study and the literature [6, 13, 16, 17] all similarly reported that LBP had the highest prevalence rate, that there was a decrease in the prevalence rates in all the studies for seeking medical care, and that the lowest prevalence rate was for lost time due to LBP. Interestingly, two sites in this current study, UU and TAMU, had higher prevalence for lost time due to LBP than seeking medical care for LBP, which contradicts the literature and the aforementioned theory of progression. These differences may be due to a combination of workers compensation insurance differences and/or cultural differences in seeking medical care for workplace injuries. The UU and TAMU pattern may also be due to factors not studied. One possible explanation for the lack of seeking care in Utah and Texas may be the lack of available health care providers in the region. Combs et al. [21] reported that the ratio of primary care physician to population, which is how primary care physician availability has been evaluated, was 63.4 in the state of Utah in 2008, which was the lowest among all 50 states. Gong et al. [22] found Texas to be one of the states with a severe physician shortage between 1999 and 2004. The American Association of Medical Colleges in 2007 ranked all 50 states from highest to lowest on the ratio of physician to population, Texas and Utah were among the bottom 10 states on the ratio [23]. Thus it was thought the low prevalence for seeking medical care in the UU and TAMU data may be due to the lack of availability of physicians in the regions where those data were collected. Alternatively, there may be sufficient physicians in the region for healthcare needs and instead some cultural differences not captured by ethnicity or other questions that promote self-care instead of seeking care from a medical provider in the Utah and Texas regions, which may result in lower prevalence for seeking care compared to lost time due to LBP. Regardless, the overall trend in the current study followed the pattern established in the literature, which illustrates the strength of having a large and diverse sample.

The 12-month period prevalence rate of LBP lasting 7 days or more in the current study was 25%. Both Abolfotough [13] and Feng [17] reported prevalence rates much higher than the current study. It is hypothesized that the higher prevalence in the literature are due to the one-day duration of symptoms compared to the 7-day duration with the current study. This hypothesis is supported by Ozgular et al. [6], which reported a 1 day LBP prevalence of 43% as well as a prevalence rate of 17% for LBP lasting 30 days. Thus, for the shorter duration of pain symptoms the prevalence rate was higher than the current study and for the longer 30 day duration of symptoms the prevalence was lower than the current study.

In the current study, the seeking medical care for LBP definition had a 12-month period prevalence of 14%. This prevalence rate is low in comparison to the literature, which ranges from 17 to 38%. In the report ranking the ratio of physician to population [23] by state, Ohio and Michigan are at the average with 236.6 physicians per 100,000 population and rank 17 and 18, respectively. The state of Illinois ranks 21, Wisconsin ranks 24. Thus none of the participating states in the current study rank particularly high on the physician to population ratio scale. This may be one explanation for why the seeking care prevalence was lower in the current study compared to the literature. The Merlino study was completed in the United States with 17% prevalence. The Merlino et al. [16] was collected in Iowa, Illinois, Oregon and Washington, which rank 41, 21, 13 and 16, respectively on the number of physicians per 100,000 in population in 2007. It may be that the increased availability in physicians in Oregon and Washington resulted in an increased prevalence in the Merlino study compared to the current study since both studies examined manual material handling workers. The higher prevalence rates for seeking medical of 38% [17] and 34% [13] may also be due to the population studied. It is hypothesized that nurses or health care providers may be more likely to seek medical care for LBP than those who perform manual material handling tasks.

Our analyses indicate that there were significant differences among the sites for seeking medical care. In a study of the American population seeking medical care for various illnesses, it was found that race/ethnicity significantly influenced seeking care for LBP [10]. The current study did not show a statistically significant difference in seeking medical care due to race/ethnicity nor was there an interaction between the sites and race. In a study examining ICD-9 codes for pain, the United States was divided into 4 regions including south, west, north central and northeast [24]. The two ICD-9 codes that were most comparable to the current study were LBP and degenerative disc disease codes. Interestingly, there were significant differences in prevalence in each of these ICD-9 codes among the different regions of the country. Different sites in the current study collected data from different states. The seeking medical care for LBP measure had higher 12-month period prevalence rates in Ohio and Michigan compared to Illinois, Wisconsin, Utah and Texas. The regions defined by Murphy et al. [24] do not match up exactly to the states in the current study however the common finding between the two studies is a statistically significant difference in 12-month period prevalence for seeking medical care as a function of the state or region within the United States.

**Fig. 3:**
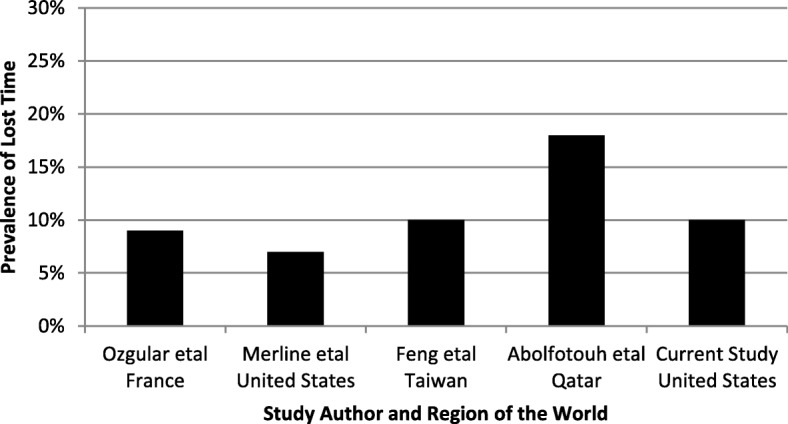
Prevalence of lost time as a function of study author and region of the world where data was collected

The third 12-months prevalence measure was lost time and is thought to indicate the most severe level of low back health effect quantified. Overall, the current study had a lost time prevalence of 10%. Ozgular et al. [6] had a prevalence rate of 9% for sick leave or lost time, which was very close to the current study. Merlino et al. [16] reported a lost time 12-month period prevalence rate of 7%, which was slightly lower than the current study. Feng et al. [17] found a sick leave 12-month period prevalence of 10%, the same as the current study. Abolfotuoh et al. [13] reported a 12-month period prevalence of 18% for lost time, which was slightly higher than the current study. Given the differences in the populations studied, the job exposures and the variability in the LBP prevalence among these studies it is remarkable that the lost time prevalence among these studies is this comparable. Figure 3 illustrates the consistency in the prevalence of lost time among the different studies with various populations and across different cultures, suggesting that this may be a stable surveillance measure for study of low back health effects. Davis and Kotowski [11] in a review of musculoskeletal disorders among nurses found that prevalence of self-reported lost time was the least investigated low back health surveillance measure. Given the stability of the self-reported lost time surveillance measure in the literature, future research may want to focus on self-reported lost time instead of pain surveillance measures. It should be noted that this was a measure of self-reported lost time away from work and not disability. In some cases, disability has been measured via a subjective questionnaire to quantify impairment of activities of daily living and not a measure of work disability [25]. Self-reported lost time from work may provide an effective and consistent surveillance measure that eliminates some of the confounding factors that may influence subjective disability and pain measures.

Examining the 12-month period prevalence of LBP lasting at least 1 week, seeking medical care for LBP, and lost time due to LBP may provide a mechanism to quantify low back health effects that suggests evidence of a possible progressive disease leading to disability, as theorized by Ferguson and Marras [15]. With this theory, the least severe measure would be LBP of any duration, and would more likely be studied through population-based questionnaire data. Interestingly, prevalence of LBP may be as low as 4% for chronic LBP [2] and as high as 69% for 1 day LBP in Malaysian railroad workers [4]. Seeking medical care for LBP prevalence rates ranged from 4.5% [7] to 32% [8] and lost time, the most severe measure of LBP prevalence rates, ranged from 4.6% [12] to 18% [13]. Thus, the more severe the definition of low back health effect used, the smaller the range and average prevalence in the literature. If LBP is a progressive disease process, then by examining the exposure-response relationships between the risk factors and each low back health effect measure we may develop a greater understanding of the interactions of physical, psychosocial and psychological components at each stage of the process and potentially reduce the risk of the progression. More research that examines the progression of LBP measures in individuals across a spectrum of low back health effects would enhance our understanding of whether, and to what extent, there is a progression of LBP leading to disability. In addition, research into the progression latency of the LBP measures may help management and insurance companies plan strategies for reducing incidences and costs of LBP.

### Limitations

There are several limitations in this study. First, it only examined 12 month period prevalence of LBP lasting 7 days, seeking medical care due to LBP, and lost work time due to LBP and the difference among the definitions may not be related to causal pathways. Second, this study had all manufacturing and distribution center jobs and therefore the results may not be applicable to other workplace settings. Third, the study population was predominantly male therefore the study results may not extrapolate to majority female populations. Finally, this paper examined only a portion of a large data set, the inclusion of physical job demands and workplace psychosocial factors in future analyses may help control some of the potential confounding effects of regional differences on various measures of low back health outcomes.

## Conclusions

The 12 month period prevalence for low back pain lasting at least 1 week, medical care due to LBP and lost work time due to LBP was 25, 14 and 10%, respectively. There were significant differences in period prevalence rates among the sites or regions of the United State for seeking medical care as well as lost time due to LBP.

## Abbreviations

LBP: Low Back Pain
NIOSH: National institute of Occupational Safety and Health
OSU: Ohio State University
TAMU: Texas A&M University
UU: University of Utah
UWM: University of Wisconsin-Milwaukee
